# The newly credentialed veterinary technician: perceptions, realities, and career challenges

**DOI:** 10.3389/fvets.2025.1437525

**Published:** 2025-01-17

**Authors:** Addie R. Reinhard, Victoria P. Celt, Leah E. Pilewki, Mariel K. Hendricks

**Affiliations:** ^1^MentorVet, Lexington, KY, United States; ^2^Richard A. Gillespie College of Veterinary Medicine, Lincoln Memorial University, Harrogate, TN, United States

**Keywords:** burnout, utilization, longevity, veterinary technician, veterinary wellbeing

## Abstract

Previous research has shown that when comparing psychological wellbeing between credentialed veterinary technicians and human registered nurses, high levels of burnout and distress were noted within the veterinary technician population. This qualitative focus group study sought to gain a better understanding of the early-career veterinary technician experience to understand what factors might contribute to stress and burnout. Four ninety-minute qualitative focus groups were conducted with a range of two to five participants. Semi-structured interviewing was used, and focus groups were recorded, transcribed, and then analyzed. Two major categories were identified: (1) The Credentialing Journey, and (2) Challenges with the Career. Seven themes were identified in these two categories, including Credentialing: Perceptions and Reality, the Newly Credentialed Veterinary Technician, Us vs. Us, Ethical Dilemmas, Work Environment, Utilization, and Career Longevity. The findings of this study can inform future intervention research to improve the mental health and wellbeing of veterinary technicians. A focus on teaching team-based medicine, leadership skills, and conflict management in both veterinary and veterinary technician schools may help reduce team conflict. In addition, effectively utilizing veterinary technicians and increasing awareness of their value may help improve veterinary technician career satisfaction.

## Introduction

Veterinary technicians, also known internationally as veterinary nurses, are an integral part of the veterinary practice and are trained to assist veterinarians in the care and handling of animals. Their role involves providing technical support in areas such as anesthesia, providing nursing care, diagnostics, client education, and surgical assisting (1). In the early 1960s, the first veterinary technology programs were established formalizing their role and providing structured training on skills necessary to support high-quality veterinary care (2). Typically, veterinary technology programs include around two to four years of training after high school, and to become licensed, individuals must pass a credentialing examination (1). Their advanced training and expertise have been recorded to positively impact gross practice revenue (3, 4).

Despite the valuable contribution veterinary technicians make to both the financial success and the operational stability of a practice, it has been shown that when comparing the psychological wellbeing of veterinary technicians to normative samples of human registered nurses (an analogous group), high levels of psychological strain and burnout are prevalent in this profession (5).

The presence of burnout has been researched throughout the veterinary profession with one study citing that 58.6% of veterinary technicians surveyed scored above the burnout threshold (6). Burnout occurs when there is prolonged workplace stress and is often accompanied by emotional exhaustion (7). Veterinary workplace occupational stressors have also been correlated with poor psychological health and physical health. In the veterinary professional workplace, stress has been linked to emotional exhaustion, cynicism, and intention to leave (8).

There are numerous unique occupational stressors that veterinary technicians experience that can result in emotional health problems. Research done by Foster and Maples reported that 73% of veterinary technicians were experiencing high levels of stress, and the top three reported occupational stressors were workload, death and dying, and conflict with veterinarians (9). Other research has found that high demand and low control job conditions also promote stress in the work environment and can place individuals at risk for psychological strain and burnout (10). Veterinary technicians often care for sick, hurt, and dying animals; this, coupled with animal cruelty cases as well as the consolation of grieving owners can negatively affect well-being (11). Repeated exposure to occupational stressors can also lead to compassion stress and fatigue (11). For example, an Australian study on veterinary nurses reported that 82% were experiencing compassion fatigue and 94% reported work-related stress (12).

In a profession where veterinary technicians are so valuable, it is troubling to see that among the veterinary team, technicians have some of the lowest scores for job satisfaction (13). Upon examination of factors that contributed to job satisfaction and burnout, a coordinated team environment was associated with positive results while the opposite was found in a toxic team environment (13), which indicates that team effectiveness plays a key role in job satisfaction and burnout. When examining the factors that influence job satisfaction for veterinary nurses in the UK, participants who were unhappy in their current employment reported the desire to leave due to a lack of support and communication within their practice (14).

Veterinary professionals who experience repeated stressors are vulnerable to poor mental health outcomes and perhaps even increased risk of suicide. It is estimated that male veterinary technicians are five times and female veterinary technicians are over two times more likely to die by suicide as compared to the general population (15). An Australian study found that although statistically insignificant, veterinary technicians had suicide rates higher than that of the general public (16).

Accompanying the mental stressors veterinary technicians face, physical stressors are also common. Physical restraint of patients can be taxing and lead to injuries and chronic physical pain. In a surveyed group of veterinary nurses, 98% reported acute injuries such as dog and cat bites and scratches (17), while chronic back and neck pain was experienced by 52% (17).

While current quantitative research focuses on the presence or absence of psychological wellbeing, burnout, and occupational stress in veterinary technicians, this qualitative focus group study sought to gain a better understanding of the early-career veterinary technician experience and determine what factors might contribute to stress and burnout within the career with the ultimate goal of informing future intervention research. The guiding research question of this study was:

- What are the experiences of a newly credentialed veterinary technician, including major challenges and stressors faced?

## Methods

### Participants and recruitment

This study consisted of one male and sixteen female veterinary technicians, for a total of seventeen participants. The inclusion criteria for this study were credentialed veterinary technicians over the age of eighteen years of age who had graduated within the past seven years. The ages of participants varied widely with a mean year born of 1989 and a median year born of 1993. All participants were white. Their roles in the field also varied with seven working in companion animal exclusive practice, three classified as working in industry/commercial organizations/pharmaceutical, two in college or university, one in not-for-profit, one in federal government, one in equine, one in small animal specialty, and one in mixed animal practice.

The study was approved by the Institutional Review Board at Lincoln Memorial University College of Veterinary Medicine. Veterinary technicians were recruited in partnership with the National Association of Veterinary Technicians in America (NAVTA) using email and social media recruitment. Each participant was given a pseudonym to protect their privacy and used for both the focus group and this paper.

### Research design and data analysis

Four ninety-minute qualitative focus groups were conducted on Zoom with a range of two to five participants. Semi-structured interviewing was used, and a series of open-ended questions (see Table 1) were asked regarding participants’ experiences of being a newly credentialed veterinary technician. Discussion between participants was encouraged. Focus groups were recorded, transcribed, and then analyzed. First-cycle eclectic coding which combines various first-cycle coding methods was leveraged using Delve, a qualitative coding software. In this study, a combination of in-vivo, descriptive, and attribute coding was used. Second-cycle focus coding lumped common and/or significant codes together into themes and categories of themes. Finally, the integration of keywords from coding into a narrative was leveraged to create the veterinary technician experience.

**Table 1:** Focus group questions.

Q1. Please share with us the type of veterinary practice you are working in and how long you have worked at that practice. Q2. Describe your experience transitioning from veterinary technician student to practicing veterinary technician. Q3. What aspects of the veterinary technician profession did you not feel prepared for early in your career, if any? Q4. What types of resources would have been helpful to you during your transition to practice? Q5. Please describe some of the challenges you currently face as a veterinary technician, if any. Q6. What resources would you find helpful in combating the challenges you face? Q7. What non-clinical professional skills or knowledge do you feel are most important as a veterinary technician? Q8. What non-clinical professional skills did you feel least confident in during your early career as a veterinary technician? Q9. What non-clinical professional skills do you feel least confident in now, having formal education and experience in the profession? Q10. Describe the level of connection or sense of belonging that you currently feel in the veterinary community? Q11. What advice would you give to students or new technicians transitioning into practice?

## Results

Seven themes were identified and were categorized into two major categories including the Credentialing Journey (themes identified included Credentialing: Perceptions & Reality and the Newly Credentialed Veterinary Technician) and Challenges within the Career (themes identified included Us vs. Us, Ethical Dilemmas, Work Environment, Underutilization, and Career Longevity).

### The credentialing journey

The themes identified within this category explored veterinary technicians’ motivations for credentialing, their experiences transitioning into credentialed roles, and the impact these changes had on their job duties and professional identity.

#### Credentialing: perceptions and reality

Veterinary technicians decided to get credentialed for a variety of different reasons. The most common reasons cited included the desire for a salary increase, advancing their education, and providing a higher quality of care because of understanding “the why” for what they were already doing in practice. Despite their qualifications, many participants felt misunderstood and disrespected by clients, doctors, and team members. Winona, a former small animal veterinary technician now working for a pet insurance company, mentioned that some clients were only nice to veterinarians and disrespected veterinary technicians and other team members. Josh, a mixed animal general practice technician shared,

I think that a lot of the public does not really take us seriously, especially a lot of the clients. They’re just like, they are just a technician… I do not want to talk to them. I want to talk to a doctor. Some clients even want doctors to do nail trims on their pets because they do not think that we are qualified enough to do those…. We went through all that effort to get the knowledge that we do, and then clients treat us like we do not know anything at all. That’s really hard and it’s very upsetting.

In addition, title protection which restricts the use of the title “veterinary technician” to those who meet specific education and credentialing requirements was mentioned by many participants as important. However, many shared that title protection for veterinary technicians does not exist in their states, allowing uncredentialed individuals to use the title, which can diminish the professional identity and respect for the role and has implications for patient safety.

Josh, emphasizing the importance of education and training for patient safety, explained, “Like cystos. You can very easily injure a patient if you do not know what you are doing without that education we have.” Melanie, who worked in Maryland, expressed frustration with the absence of title protection in her state, where uncredentialed individuals frequently perform tasks typically reserved for credentialed technicians. She shared her excited for moving to a state with greater title protection, stating, “I’m happy to be able to go to a place… [that] respects the education more.”

#### The newly credentialed veterinary technician

The experience during the transition into becoming credentialed varied between participants. Some technicians felt that their job roles did not change significantly after getting credentialed except for a “change in title, increase in pay.” Many felt that their responsibilities overlapped significantly with veterinary assistants in practice, or as Creed, a veterinary technician within an animal welfare role, shared, “Everybody just does it all.” While many reported no change after becoming credentialed, others were asked to assume leadership roles, including taking on new tasks such as training others in the practice. Sometimes they were met with resistance in these new roles. Sarah, an industry veterinary technician who previously worked in small animal medicine shared,

It gave me more of an opportunity to take on a leadership role and kind of guide… the assistants into more of a better way to practice and they did not appreciate that…. So that became a little bit of a struggle trying to find my footing and trying to find a way to utilize what I’ve learned in school to advance on my assistant duties and to help other people get there.

Some participants felt that their school training did not mentally prepare them for practice, and some struggled to translate both technical and emotional skills learned in school into practice, particularly during stressful situations. Trin, a small animal general practice veterinary technician, shared, “Nothing really prepares you for an emergency until you have had to do a couple.” The mental burden was intense for many. Danielle, a general practice veterinary technician, recounted,

A puppy that got into Rodenticide… this puppy just vomited a ton of blood and then just started to arrest. So that was pretty traumatic for me. I knew about all about cases like that in school and everything, but just like in real life… it’s just something that I do not even know if you can prepare for.

### Challenges within the career

Building on the experiences of becoming credentialed, the following themes highlight challenges that veterinary technicians face in their careers. These include barriers and stressors that affect day-to-day work, such as team conflict, ethical dilemmas, work environments, underutilization, and concerns about career longevity.

### Us vs. Us

Team conflict was one of the most commonly mentioned stressors and challenges, including conflict while working with assistants, veterinarians, and other credentialed technicians. Conflict with veterinary assistants was mentioned several times during the focus groups, particularly related to training and development, the overlapping roles and responsibilities of the two job positions, and bullying. Beth, an emergency and critical care veterinary technician, shared, “hospital management saying… ‘you went to school for this, and now you can teach this person that we just grabbed off the street how to do your job.’” Bullying was mentioned as a significant stressor for Trisha, a general practice small animal veterinary technician, who shared she was seeking therapy because “a girl in our clinic that would constantly put down other people… it was to the point where… I’m not going to place a catheter right now because I cannot keep my hands still because I’m so anxious.” Alice mentioned,

And then the biggest conflict I’ve probably had with assistants is overstepping. At one clinic I worked at, they did not have very many techs, so they did allow the assistants to do a lot more than they probably should have allowed, still being legal and everything. But certain assistants took that to the point of, it’s not just, I can do this when no one else is available. It became, I can do this whenever I want to.

Conflict with veterinarians was also mentioned several times by focus group participants. Creed highlighted this by sharing, “basically if a catheter wasn’t placed on the first try… they would speak to another vet in the same room and just make derogatory comments pretty regularly.” Sarah mentioned working with both supportive veterinarians and difficult veterinarians, stating,

Some veterinarians that I worked under take time out, want to help me really advance on certain skills or sit down and help educate me… then I’ve had some veterinarians who have screamed at me and pointed their finger at me like a drill sergeant if I did not get the x-ray positioned exactly right or if I did not give the vaccine with the correct size needle that they wanted… I have gone nose to nose, literally with a veterinarian. He was screaming in my face, and I finally had enough and I screamed back.

Conflict was also mentioned between credentialed veterinary technicians at practices. Catherine, a veterinary technician working in telehealth, discussed conflict when trying to implement new protocols or new ideas in the practice, sharing that many of the credentialed technicians who had been in the field for a long time were not open to change.

### Ethical dilemmas

Ethical dilemmas and moral stress were mentioned by several participants as a challenge faced. Josh, a veterinary technician working in mixed animal general practice, mentioned frequent exposure to animal abuse, sharing, “people here do not take care of their pets very well. There’s a lot of cruelty cases that I deal with” and described a hoarding case that was “really hard to deal with.” Trin shared,

It was severely emaciated dog apparently been seizing all night…. And it took them 3 h to decide to finally euthanize. And I got to stay with that dog the entire 3 h with people occasionally checking on me…. I went home and I just cried and I was like, I do not even know if I want to do this…. I could not really do anything besides just hold this animal and watch her die…. I was just sitting there going, please, somebody make a decision or take this away from me.

Kathy, a small animal and exotic technician, described the challenge of witnessing ethical dilemmas occurring while simultaneously feeling as if she had no power to alter the situation. She shared,

I often see cases come in where people have just let the animals suffer for too long. And my vet will rarely say, I think it’s time…. And the animal winds up coming back a couple of days later and being euthanized or a DOA, and it really disturbs me. I want to speak up, but it’s not my place.

During the focus groups, some veterinary technicians voiced concerns regarding veterinary assistant utilization in the practice. Leveraging veterinary assistants to perform certain tasks such as anesthesia was sometimes perceived as unsafe or dangerous. Winona, who started her career as a veterinary assistant before getting credentialed, shared, “Oh my God, if clients knew that some hospitals have assistants doing those things. I did not know anything. I worked in the clinic for six weeks, and now, I’m monitoring anesthesia.” Sarah shared, “many people run… anesthesia for patients and know nothing about what they are doing… it’s scary.”

### Work environment

A common theme that was identified during this study was the impact of the work environment on veterinary technicians in the focus group. Many veterinary technicians discussed the challenges of navigating issues with practice management and coping with being short-staffed. Management issues were mentioned by three veterinary technicians and two participants mentioned that management favored certain team members. However, Trisha mentioned management at another clinic was extremely fair and helpful to staff when dealing with clients, including advocating for the veterinary team and setting boundaries with clients when appropriate. When leaders in the practice did not set healthy boundaries with clients, this had the potential to impact the rest of the team. Winona shared,

And it was really hard for me to get our doctor who owned the practice, to basically put our foot down that we will not tolerate certain behavior… having clients come into a hospital… rude to your receptionist, and then they are rude to your tech, but they are nice to the doctor… you have to put your foot down when clients are stepping out of line.

### Utilization

Another theme mentioned ten times through the four focus groups related to utilization. Many veterinary technicians in the focus group felt that they were being underutilized for skills they were trained for in school and often being overburdened with tasks that “pretty much a warm body, any warm body could do.” Sarah highlighted this theme by sharing, “I was being underutilized but expected to do a lot of things at the same time, if that makes sense. Like running with your head cut off but do not get to use your actual skills.” Alice, who works both as a small animal and equine veterinary technician, shared,

There’s things that I’ve learned and things that I’m legally allowed to do, but I’m not allowed to do them. That vet’s either do not let me do them or our local association has said no, techs aren’t actually allowed to do that…. You’re throwing everything else on me except the stuff that I’m allowed to do.

Though focus group participants wanted to be utilized to perform the skills they were trained for, the willingness of veterinarians to help when needed, particularly if the technician was busy, was viewed as important. Beth highlighted this delicate balance of utilization, sharing,

Having doctors pitch in because… they’ll come up and be like, “hey, can you draw blood on this dog?” And like, you are also capable of drawing blood. You can do that while I’m doing, you know, 20 patients over here… you can use your common sense and help out when I’m slammed.

Only one technician in the study mentioned being properly and effectively utilized.

### Career longevity

Career longevity was discussed as a concern for many of the focus group participants. Some believed a long-term career as a veterinary technician was not feasible. The physical and mental demands of the career were frequently mentioned. Participants mentioned extremely long shifts, handling large or difficult patients, and navigating burnout as contributors to an unsustainable career path. Concerns regarding career advancement and career flexibility were also discussed by several focus group participants. Catherine mentioned challenges obtaining job duty alternatives within the practice as it became difficult to practice if one had physical impairments,

I was having health issues with my hands… it is physically unsafe for me to do some of these care tasks, but there’s not really a helpful route of maintaining your credentialed pay.

Lydia described feelings of frustration over the lack of career advancement opportunities, stating that she loved emergency medicine and her job, but “then it’s discouraging because it’s supposed to be the top of where you can go to do this kind of thing. So it’s like, well, where do you go from here?”

## Discussion

Veterinary technicians face many stressors within their careers, and this study aimed to gain a deeper understanding of the experiences of newly credentialed veterinary technicians using qualitative methods. In four focus groups of seventeen veterinary technicians total, seven themes included: Credentialing: Perceptions and Reality; The Newly Credentialed Veterinary Technician; Us vs. Us; Ethical Dilemmas; Work Environment; Utilization; and Career Longevity.

Conflict in the veterinary workplace is common and has been described as a key stress factor (18). Similarly, in this present study, the Us vs. Us theme was identified as many veterinary technicians in the focus group described conflict among the team as challenging. Several techniques can be utilized to support veterinary technicians facing team conflict, including conflict management training, leadership training, as well as a focus on team-based medicine in all formalized veterinary professional training. Team-based care is considered a collaborative approach to providing healthcare in which all team members work together valuing the unique knowledge and skills of each individual on the team (19). In human medicine, this approach to delivering care has shown a significant impact on patient and economic outcomes with improved efficiency, increased job satisfaction, and better service quality (20).

Many veterinary technicians in the focus group described having a work environment that was not supportive, leading to additional challenges for newly credentialed technicians. Veterinary hospital leaders should consider seeking opportunities for leadership training to better understand how to create more supportive work environments. The Merck Animal Health Veterinary Wellbeing Study found that having a supportive work environment in which individuals felt a sense of trust within the organization and a strong sense of belongingness to the team with candid and open communication was one of the most important predictors for good mental health and wellbeing within the profession (21).

In the focus group, a theme of utilization was identified. Many veterinary technicians felt dissatisfied when they were overworked for certain tasks that anyone could do and underutilized for the technical skills they learned in school. Effective utilization of veterinary technicians has been shown to increase practice profitability by over $93,000 according to the 2009 AVMA Report on Veterinary Business Measures (4). A Canadian study found that for each additional credentialed technician per veterinarian, the revenue per veterinarian within a year increased by over $75,000 (21). In addition, in a recent NAVTA membership study, over half of veterinary technicians felt they were not or only sometimes utilized to their full potential within their practice (22). Educational strategies in continuing education or in veterinary schools to create broader awareness of veterinary technician skills may help to improve effective and appropriate utilization. In addition, developing protocols within hospitals to effectively utilize veterinary technicians may not only improve practice profitability but also veterinary technician satisfaction in the workplace.

Finding career longevity is a multifaceted process. Technicians are in a physically demanding job position and taking care of their physical bodies is critical to career sustainability. Educational strategies within hospitals encouraging healthy lifting, promoting physical wellness (e.g., providing a gym membership as part of employee benefits), and sharing resources (e.g., providing articles on creating physical fitness routines) (23) may help technicians stay in veterinary medicine long term. In addition, alternative career opportunities are available if a technician finds clinical work to be too physically demanding including positions within research, education, industry, etc.

Title protection was also mentioned as a key stressor for credentialed veterinary technicians in the current study. In 2022, research reported by NAVTA showed only eleven states define the title “veterinary technician” and only five states have criminal penalties for individuals misrepresenting themselves as veterinary technicians (22). The widespread lack of title protection can result in many credentialed technicians feeling undervalued and disrespected (22). Veterinarians and team members should make efforts to correctly identify titles being used within their practices. NAVTA also recommended that educational institutions stress the importance of proper identification within teaching programs and educate both veterinary and veterinary technician students on laws and regulations surrounding the title of a credentialed veterinary technician (22). Grassroots efforts for legislative changes, being led by state veterinary technician associations, are also occurring in many areas of the country.

The main limitation of the present study is the lack of racial, ethnic, and gender diversity. All veterinary technicians in this study were white and 94% were female. Referencing national demographic statistics, the 2022 NAVTA Demographic Survey identified 90% of credentialed technicians as white and 91% as female (24). This may limit the generalizability of this data across the entire veterinary technician population. In addition, due to the nature of the focus group methodology, individuals may be conformity-biased to respond in a certain way depending on how others in the group respond to a question.

Historically, veterinary technicians have stayed in their careers for five to seven years (25). As identified in this study, several stressors were discussed that would negatively impact mental health and wellbeing which may impede career longevity. By identifying these concerns and focusing on improving leadership skills, fostering team-based medicine, providing conflict management training, effectively utilizing technicians’ skills, understanding physical self-care, and increasing awareness of appropriate title usage, this study could inform future interventions on mental health and wellbeing within the credentialed veterinary technician community.

## Data Availability

The datasets presented in this article are not readily available because data contain potentially identifying information. Requests to access the datasets should be directed to AR, addiereinhard@gmail.com.
